# Multi-Task Model for Esophageal Lesion Analysis Using Endoscopic Images: Classification with Image Retrieval and Segmentation with Attention

**DOI:** 10.3390/s22010283

**Published:** 2021-12-31

**Authors:** Xiaoyuan Yu, Suigu Tang, Chak Fong Cheang, Hon Ho Yu, I Cheong Choi

**Affiliations:** 1 Faculty of Information Technology, Macau University of Science and Technology, Taipa, Macau; 1709853eii30001@student.must.edu.mo (X.Y.); 2009853gii30001@student.must.edu.mo (S.T.); 2 Kiang Wu Hospital, Santo António, Macau; alexchoi0@gmail.com

**Keywords:** classification, image retrieval, segmentation, multi-task, esophageal endoscopic images

## Abstract

The automatic analysis of endoscopic images to assist endoscopists in accurately identifying the types and locations of esophageal lesions remains a challenge. In this paper, we propose a novel multi-task deep learning model for automatic diagnosis, which does not simply replace the role of endoscopists in decision making, because endoscopists are expected to correct the false results predicted by the diagnosis system if more supporting information is provided. In order to help endoscopists improve the diagnosis accuracy in identifying the types of lesions, an image retrieval module is added in the classification task to provide an additional confidence level of the predicted types of esophageal lesions. In addition, a mutual attention module is added in the segmentation task to improve its performance in determining the locations of esophageal lesions. The proposed model is evaluated and compared with other deep learning models using a dataset of 1003 endoscopic images, including 290 esophageal cancer, 473 esophagitis, and 240 normal. The experimental results show the promising performance of our model with a high accuracy of 96.76% for the classification and a Dice coefficient of 82.47% for the segmentation. Consequently, the proposed multi-task deep learning model can be an effective tool to help endoscopists in judging esophageal lesions.

## 1. Introduction

The incidence of esophageal lesions is getting higher and higher due to the continuous growth of the population and the changes in the lifestyles of people. Especially, esophageal cancer is the tenth-most common cancer in the world and has the sixth-highest mortality rate [1]. Fortunately, the treatment of esophageal cancer benefits from early detection; that is, it has a 5-year relative survival rate of more than 90%, while the later survival rate is less than 20% [2]. It also brings great troubles to the health of people.

At present, the typical strategies currently used to detect esophageal lesions are gastrointestinal endoscopic screening such as white light imaging (WLI), narrow-band imaging (NBI), capsule endoscopy, and so on. Unfortunately, there are some deficiencies in using gastrointestinal endoscopic to diagnose esophageal diseases. It is difficult to accurately diagnose patients with esophageal cancer based on WLI because of its lower sensitivity and specificity [3]. NBI not only requires experienced endoscopists to perform operations but is also expensive for examinations [4]. Therefore, less-experienced endoscopists are more likely to be unable to distinguish similar esophageal lesions and there is a lack of NBI equipment in low-income countries or regions. Likewise, capsule endoscopy will produce a large number of esophageal images. The endoscopist needs to make a diagnosis within a limited time, find the diseased regions, mark them, and finally, determine the treatment schemes [5]. In other words, endoscopists are busy fighting esophageal diseases every day. As a result, the manual diagnosis process based on gastrointestinal endoscopic screening of the esophagus is affected by many negative factors, such as the experience level and mental state of endoscopists, the limitation of the diagnosis time, the huge esophageal image base, the subjective differences of different endoscopists, and so on. This is why the diagnosis of clinical esophageal lesions still has a high rate of missed diagnosis and misdiagnosis [6,7]. Therefore, it is of great significance to develop a support tool based on deep learning to not only classify but segment the lesions in esophageal endoscopic images, so as to reduce the burden on endoscopists, thereby improving the diagnosis accuracy.

Recently, benefiting from the rapid development of deep learning, many advanced techniques based on deep learning have been applied in various medical fields, such as skin lesion segmentation [8], retinal blood vessels segmentation [9], prostate cancer analysis [10], etc. As for the esophageal lesions analysis, such deep learning approaches have shown success in object detection [11], image classification [12], and semantic segmentation [13].

Although deep learning methods have made great contributions, especially for those diseases with a high fatality rate or difficult to diagnose, most of them have a common problem that they only focus on a given task or a certain type of disease, such as esophageal squamous cell carcinoma [14]. Therefore, it is very necessary to use deep learning methods to do a comprehensive analysis instead of only targeting a certain disease. Moreover, the aid information they provide to endoscopists is also limited, usually to only one classification accuracy rate. Especially for difficult-to-diagnose or controversial samples, endoscopists should be provided with more effective aid information, not just a simple classification accuracy rate. In other words, a multi-task model based on deep learning can provide endoscopists with various aid information in clinical applications, thereby making a diagnosis more efficient and more accurate. For example, esophageal lesion classification first distinguishes the types of esophageal lesions, and then, esophageal lesion segmentation can further determine the lesion regions. If the deep learning-based classification and segmentation models are developed separately, this consumes a long training time and requires large storage to store all the subnetwork models.

To solve the above problems, developing a model to achieve multiple tasks is a good strategy by using the shared features between different single-task deep learning models. Multi-task learning is an important paradigm of deep learning. Its goal is to mine common features between different tasks to improve the performance of the model and its better generalization ability [15]. The basic idea of multi-tasking is that different tasks can share some common features, so they are jointly trained. There are two methods commonly used in multi-task learning based on convolutional neural networks: soft parameter sharing [16] and hard parameter sharing [17]. Soft parameter sharing designs a model for each task with its parameters and uses regularization as a constraint to realize parameter similarities. Hard parameter sharing is to share the same hidden layers of the model between multiple tasks but have different task layers of the model to implement different tasks. It is noted that hard parameter sharing is the most common method of multi-task learning in neural networks, which can be traced back to the literature [18].

Therefore, based on the hard parameter sharing of multi-task learning, we developed a multi-task deep learning model that achieves classification and segmentation for esophageal lesions using endoscopic images at the same time. The classification task no longer only focuses on the prediction of a certain type of disease but can also predict esophagitis, esophageal cancer, or normal images. Additionally, image retrieval in the classification task is used to provide more aid in diagnostic information. For each query image, it can find the five most similar images from the historical patient libraries. When the endoscopists encounter a controversial or difficult-to-diagnose sample, the retrieval can provide more aid information besides classification results. The segmentation task can locate the cancer lesion area. It is better than the methods based on detection, because it avoids the problem of inaccurate positioning but a high confidence level. To achieve a better segmentation performance, we designed a mutual attention module to capture more diverse features in the segmentation task.

In summary, our contributions are mainly the following four:

(1) We proposed a novel multi-task deep learning model for automatic esophageal lesion analysis. It can synchronously achieve multiple tasks, including classification and segmentation for esophageal lesions.
(2) To provide endoscopists with more supporting information in classification, we built a retrieval module on the classification branch to assign a confidence for each prediction result. Classification and retrieval can be optimized at the same time without affecting each other.
(3) To improve the performance of esophageal cancer segmentation, we designed a mutual attention module in the segmentation task that can generate weight matrices from different features and guide each other to obtain diversified features.
(4) The experiments show that the proposed model is better than other similar methods and can effectively help endoscopists improve the accuracy of a diagnosis.

## 2. Related Works

In this section, we discuss three types of works that are most related to our work, including esophagus classification, esophagus segmentation, and a multi-task medical image analysis.

### 2.1. Esophagus Classification

A lot of the traditional classification method was proposed to classify esophageal lesions based on color and texture information. For example, Munzenmayer et al. [19] proposed a method based on a color texture analysis in a content-based image retrieval framework for precancerous lesions classification. Riaz et al. [20] put forward the autocorrelation Gabor feature method to extract the texture features of gastroenterology imaging for gastroenterological classification (normal, precancerous, and cancerous) and achieve better performance. Additionally, some machine learning algorithms, such as support vector machines [21] and principal component analysis [22], were employed in esophageal lesion classification.

Instead of the traditional classification methods, the deep learning-based method has been used in the classification of esophageal disease using endoscopic images. Kumagai et al. [23] constructed a GoogLeNet-based artificial intelligence tool to distinguish malignant and nonmalignant esophageal squamous cell carcinoma. Liu et al. [24] brought forward a transfer learning framework by fine-tuning pretrained models, such as VGGNets, Inception, and ResNets, to successfully classify gastric images into chronic gastritis, low-grade neoplasia, and early gastric cancer. Du et al. [25] proposed an efficient channel attention deep dense convolutional neural network that can classify diseases into four categories with a higher area under the curve value. We can see that the above-mentioned deep learning models could achieve obvious success in esophagus classification. An overview comparison of the methods for esophageal lesion classification is shown in Table 1.

### 2.2. Esophagus Segmentation

Many efforts are devoted to addressing esophagus segmentation by developing effective methods. Before deep learning, most methods used shape or appearance models to guide esophagus segmentation [27,28]. However, this model is difficult to train and has poor robustness. Sommen et al. [29] then proposed the algorithm that computes local color and texture features based on the original and the Gabor-filtered image to annotate regions of early esophageal cancer. Furthermore, Yang et al. [30] proposed an online atlas selection approach to choose a subset of optimal atlases for automatic segmentation of the esophagus.

Inspiring by the successful application of deep learning-related methods in esophagus classification, a growing number of deep learning-based approaches have been used in esophagus segmentation. For instance, Mendel et al. [31] used convolutional neural networks based on pretrained ResNets by a transfer learning method to segment adenocarcinoma in Barrett’s esophagus. With the great success of U-Net in medical image segmentation [32], many of its variants have been proposed to employ esophagus segmentation. Huang et al. [33] proposed channel attention U-Net to segment esophageal cancer with a higher Dice value. Tran et al. [34] proposed a novel U-Net with an attention mechanism combined and STA-PLEalgorithm to achieve esophagus segmentation using 3D images. An overview comparison of the methods for esophageal lesion segmentation is shown in Table 2.

### 2.3. Multi-Task in Medical Image Analysis

Although the above-mentioned deep learning-related methods have achieved significant results in esophagus classification or segmentation, they can only achieve a given task. There are few methods that simultaneously realize classification and segmentation for esophageal endoscopic images. Wu et al. [37] proposed an esophageal lesion network composed of a classification and segmentation network for the classification and segmentation of esophageal lesions, but the classification and segmentation networks were required to be trained separately. For other medical images, multi-learning methods have been used. Chakravarty et al. [38] presented a multi-task convolutional neural network combining appearance features and structural features to achieve the segmentation and classification of glaucoma. In order to analyze skin lesions, especially melanoma, the author in Reference [39] proposed a multi-task framework to achieve three tasks: detection, classification, and segmentation. Zhang et al. [40] proposed a 3D multi-attention guided multi-task learning network by visual attention and adaptive spatial attention for simultaneous gastric tumor segmentation and lymph node classification. There are not many multi-task models applied to esophageal lesions, and they also have poor performance. The information they provide to endoscopists about each task is only a simple accuracy or Dice. Therefore, it is necessary to design a new multi-task deep learning model that can provide more aid information for esophageal lesion analysis.

## 3. Proposed Methods

In this section, we will first introduce the multi-task deep learning model as a whole and then describe each subtask in detail separately.

### 3.1. Network Architecture of The Proposed Multi-Task Deep Learning Model

**Figure 1:**
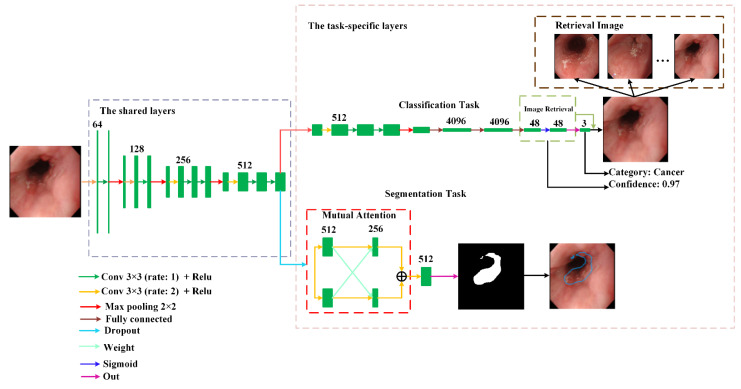
The network architecture of the proposed multi-task deep learning model.

In order to provide richer and more effective diagnostic information, we used hard parameter sharing to develop a novel multi-task deep learning model that realizes two tasks. The first task is to distinguish whether this sample is cancer, esophagitis, or normal. Additionally, based on the features of classification, image retrieval was used to find a group of images that are the most similar to the input images. The second task is to determine the lesion area when the image is cancer. Figure 1 depicts the architecture of the proposed model.

It can be observed from Figure 1 that the proposed model is made up of the shared layers and task-specific layers. The shared layers located at the bottom of the model aim to extract common features between different tasks. The task-specific layers located at the upper region of the model consist of each task branch. Since the common features in the shared layers are not suitable for direct use in each task, convolution in the task-specific layers is used to extract the features suitable for each task to improve their performance.

At last, in order to reduce the negative impact caused by the imbalance of sample categories, the classification task adopts Focal loss [41] as the loss function. It is given by:

(1) $$L_{cls}=-\alpha_{pred}\left(1-p_{pred}{)}^{\gamma }log(p_{pred}\right)$$

where γ is the focusing parameter, and γ is 2, α is 0.25.

For the segmentation task, the cross-entropy loss function is used as the loss function. It is given by:

(2) $$L_{seg}=-\frac{1}{K}\overset{K}{\underset{k=1}{\sum }}\left(g_{n}log(p_{n})+\left(1-g_{n}\right)log(1-p_{n})\right)$$

where K is the number of datasets, g is the truth label, and p is the output of the proposed model.

### 3.2. Classification and Segmentation Tasks

#### 3.2.1. The Classification Task

The goal of the first task proposed is to determine the type of input image (cancer, esophagitis, or normal). This can be obtained through the classification branch. To help endoscopists make a more accurate diagnosis, we introduced a deep retrieval module [42] to provide more helpful information. This deep retrieval module consisted of a hash coding layer and a binary coding layer. The hash coding layer, which was a fully connected layer, was used to squeeze the feature into a fixed-length hash code. It was used to reduce the computational cost of image retrieval. The binary coding layer, which limits the characteristic parameters to 0 or 1, was aimed to binarize the hash code. For each image of the training set, the deep retrieval module outputted the corresponding binary hash code as its signature. We then used these signatures to build a feature library. When retrieving, every image query will get a signature from the deep retrieval module. By similarity calculations, we found a similar image ranking to the query from the feature library. Note that, since the feature is the binarized code, we adopted the appropriate Hamming distance as the similarity assessment. Next, we utilized image ranking to compute the confidence level of the predicted result.

In the training set, the number of samples for each category is different. When we obtain the predicted category and ranking of the query through the system, we take the top-n features from the ranking as candidates. n refers to the number of samples of the predicted category. Then, the missed candidates whose category is different from the predicted category of the query are removed from the candidates, leaving k hit candidates. Finally, we use the similarity of *k* hit candidates to average all *n* candidates as the confidence level of the prediction. The confidence level can be defined as follows:

(3) $$C_{q}=\frac{\sum_{i=1}^{k}S_{k}}{n}$$

where *C_q_* indicates the confidence level of the prediction. *S_k_* means the similarity of the *k_th_* hit candidate.

#### 3.2.2. The Segmentation Task

When it was determined that the input image was a cancer lesion, the proposed model can mark where the lesion area was by the segmentation task. Compared with other detection-based methods that can only mark the approximate area of the lesion, Transformer [43] showed excellent performance in various visual fields. Inspired by SegFormer [44], we proposed a mutual attention module that can mark the lesion area more accurately, as shown in Figure 1. To enable our mutual attention module to capture different features, we used the dropout layer to generate differentiated feature maps. Then, the feature maps were fused using concatenation.

## 4. Experiments and Discussion

### 4.1. Dataset

**Figure 2:**
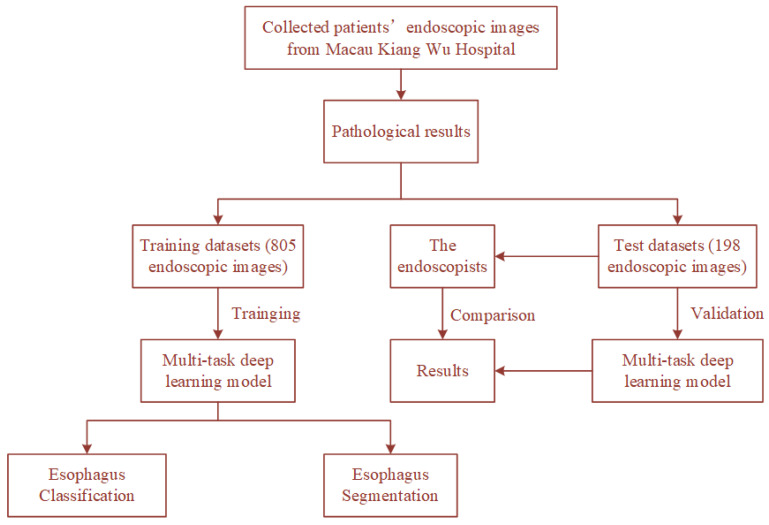
The processes of training and testing the proposed model using the dataset.

The dataset used in this study contains 1003 upper gastrointestinal endoscopy images from Kiang Wu Hospital. All images can be categorized into three classes (240 normal, 473 esophagitis, and 290 esophagus cancer). Among them, the training set has 805 images (193 normal, 379 esophagitis, and 233 esophagus cancer), and the testing set has 198 images (47 normal, 94 esophagitis, and 57 esophagus cancer). All images have pathology reports, and the lesion areas are marked by experienced endoscopists. For data augmentation, we adopt random crop, random rotation between 45 and 135 degrees, horizontal flip, and vertical flip for the training set. This comprehensive data augmentation scheme makes the network converge better. The processes of training and testing the proposed model using the dataset are shown in Figure 2.

### 4.2. Evaluation Metric

To quantitatively analyze the performance of the proposed models, we employed the following three different metrics for two tasks.

For the classification task, we calculated the Accuracy Precision, Sensitivity, Specificity, Negative Predicted Value (NPV), and F1-score to evaluate the performance. They are defined as:

(4) $$\mathrm{Accuracy}=\frac{\sum_{c=1}^{C}\left({\mathrm{TP}}_{c}+{\mathrm{TN}}_{c}\right)}{\sum_{c=1}^{C}\left({\mathrm{TP}}_{c}+{\mathrm{TN}}_{c}+{\mathrm{FP}}_{c}+{\mathrm{FN}}_{c}\right)}\times 100\%$$

(5) $$\mathrm{Precision}=\frac{1}{C}\sum_{c=1}^{C}\frac{{\mathrm{TP}}_{c}}{{\mathrm{TP}}_{c}+{\mathrm{FP}}_{c}}\times 100\%$$

(6) $$\mathrm{Sensitivity}=\frac{1}{C}\sum_{c=1}^{C}\frac{{\mathrm{TP}}_{c}}{{\mathrm{TP}}_{c}+{\mathrm{FN}}_{c}}\times 100\%$$

(7) $$\mathrm{Specificity}=\frac{1}{C}\sum_{c=1}^{C}\frac{{\mathrm{TN}}_{c}}{{\mathrm{TN}}_{c}+{\mathrm{FP}}_{c}}\times 100\%$$

(8) $$\mathrm{NPV}=\frac{1}{C}\sum_{c=1}^{C}\frac{{\mathrm{TN}}_{c}}{{\mathrm{TN}}_{c}+{\mathrm{FN}}_{c}}\times 100\%$$

(9) $$F_{1}=2\times \frac{\mathrm{Precision}\times \mathrm{Sensitivity}}{\mathrm{Precision}+\mathrm{Sensitivity}}$$

where *C* is the number of types of esophageal lesions. TP (True Positives) means the number of positive samples is correctly classified. TN (True Negatives) means the number of negative samples is correctly classified. FP (False Positives) means the number of negative samples is wrongly classified as positive. FN (False Negatives) means the number of positive samples is wrongly classified as negative.

To evaluate the image retrieval module, we adopted a ranking criterion to evaluate the retrieval performance. Given a query *q*, we obtained a ranking of each training set image using Hamming distance as the similarity measure. The precision of the query *q* in the top *k* rankings can be defined as:

(10) $$precision@k=\frac{\sum_{i=1}^{k}hit(i)}{k}$$

where *hit*(*i*) refers to whether the query *q* is consistent with the *i_th_* image label in the ranking.

For the segmentation task, we adopted the most commonly used Dice coefficient and Intersection Over Union (IoU) as the evaluation metrics. They are defined as:

(11) $$\mathrm{Dice}=\frac{2|X\cap Y|}{\left|X|+|Y\right|}\times 100\%$$

(12) $$\mathrm{IoU}=\frac{\left|X\cap Y\right|}{\left|X\cup Y\right|}\times 100\%$$

where *X* represents the ground truth, which is masked by endoscopists, and *Y* is the segmentation region of the proposed model.

### 4.3. The Classification Results

**Figure 3:**
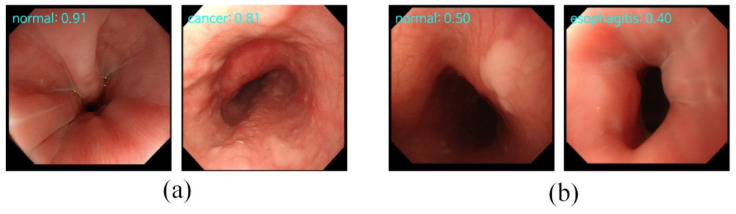
The results of classification and retrieval. “Type: 0.xx” on the top-left means the “predicted category: confidence level”. (**a**) The input images with high confidence levels indicate the high possibility of a correct prediction made by the classification task. (**b**) The input images with low confidence levels indicate the high possibility of an incorrect prediction made by the classification task.

The results of classification and retrieval are shown in Figure 3. For each image, it had a prediction given by the classification and the confidence level obtained by the retrieval. When the classification gives the correct diagnosis, the retrieval will also have a high confidence level, as shown in the first row of Figure 3. However, when the incorrect diagnosis is predicted, the confidence level will be low, as shown in the second row of Figure 3. This means that the proposed model can provide more effective supporting information when faced with difficult-to-diagnose or controversial samples. However, most of the current deep learning models just blindly improve the accuracy rate, ignoring that the main responsibility of the deep learning model is to provide effective diagnosis information.

Additionally, in order to ensure the effectiveness and robustness of the proposed model, we compared five different CNN architectures, i.e., VGG-16 [45], ResNet-18 [46], ResNeXt-50 [47], Efficientnet-B0 [48], and RegNetY-400MF [49]. These architectures were trained and evaluated with the same protocol. We first evaluated the performance of the classification task on the testing set separately. The compared results are shown in Table 3. It can be observed that the proposed model had a higher performance than the others in terms of the top-1 classification accuracy at 96.76 ± 0.22%. At the same time, we evaluated the performance of the retrieval module, and its accuracy was 91.67 ± 0.08%.

Next, to better verify the performance of the proposed model, we conducted confrontation ablation experiments on whether endoscopists refer to the results provided by the proposed model with the confidence of the predicted category. The endoscopists who participated in the testing included a senior (endoscopy experience > 10 years) and junior (endoscopy experience < 10 years), and the ratio was approximately 1:1. It is shown in Table 4.

In Table 4, we showed in detail the results of our model and the endoscopists. We can see that our model achieved the best performance on the test set. Its accuracy reached 96.96%. The precision, sensitivity, specificity, NPV, and F1 were 94.98%, 95.64%, 97.70%, 97.84%, and 95.27%. The endoscopists obtained 83.84% diagnosis accuracy without referring to the results provided by any deep learning models. Its precision, sensitivity, specificity, NPV, and F1-score were 76.41%, 78.90%, 87.90%, 87.45%, and 76.43%, respectively. To verify the effectiveness of the retrieval module, we conducted controlled experiments, as shown in Table 4. The endoscopists with a single classification indicated they only referred to the classification results. The endoscopists with our model indicated they referred to the classification and retrieval aid information provided by our model. Without the retrieval information, the average accuracy of the endoscopists was 87.26%. The average precision, sensitivity, specificity, NPV, and F1-score were 81.94%, 82.33%, 90.28%, 89.84%, and 81.30%. With the retrieval information, the average accuracy increased to 92.25%. The precision, sensitivity, specificity, NPV, and F1-score were 87.56%, 90.09%, 94.56%, 97.73%, and 82.22%, respectively. In addition, we used Cohen’s Kappa coefficient to evaluate the consistency of the diagnosis results between our model and the endoscopists. The Cohen’s Kappa coefficients between our model and endoscopists, endoscopists with single classification, and endoscopists with our model were 0.5607, 0.7048, and 0.7258, respectively. We furthermore found that, after using the retrieval module, 27 of the 38 wrong diagnoses made by the endoscopists were corrected, as shown in Table 5.

**Figure 4:**
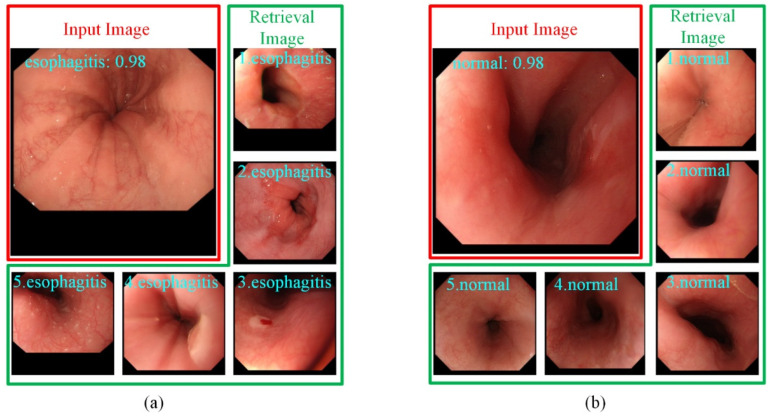
The top-5 most similar labeled samples are selected by images retrieval. (**a**) The input image is predicted as esophagitis. (**b**) The input image is predicted as normal.

Figure 4 shows the output of the retrieval images selected for the endoscopists. For each input image, besides the confidence level of the predicted category, the top-five most similar labeled images retrieved from the training set are also provided to the endoscopists for making the diagnostic decision. This additional diagnostic information is helpful for endoscopists in dealing with difficult and controversial images.

Consequently, the proposed model can not only help endoscopists improve the accuracy of diagnosis, but the additional information provided by the retrieval module can further help endoscopists make a more accurate diagnosis. This demonstrates that the proposed model can be applied to daily clinical diagnoses.

### 4.4. The Segmentation Results

Six common segmentation models were compared, including U-Net [32], PSPNet [50], FCN [51], Deeplab V3+ [52], CCNet [53], OCRNet [54], and SegFormer [44]. The Dice and IoU are shown in Table 6. We could see that the IoU and Dice of the proposed model outperformed those of other models and were 71.27% and 82.47%. Additionally, we noticed that SegFormer, which is currently the best segmentation network, achieved the second-best results. This means the attention mechanism stimulated by the transformer enabled our model to accurately locate the cancerous area.

**Figure 5:**
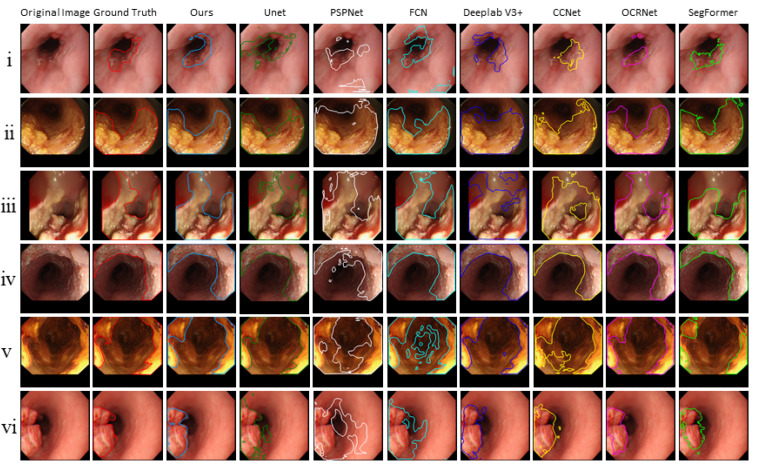
The segmentation results of the proposed model and other models.

Furthermore, the segmentation results of the proposed model and other models are shown in Figure 5. We observed that the cancer regions marked by our model were more accurate than the other models.

## 5. Discussion

**Figure 6:**
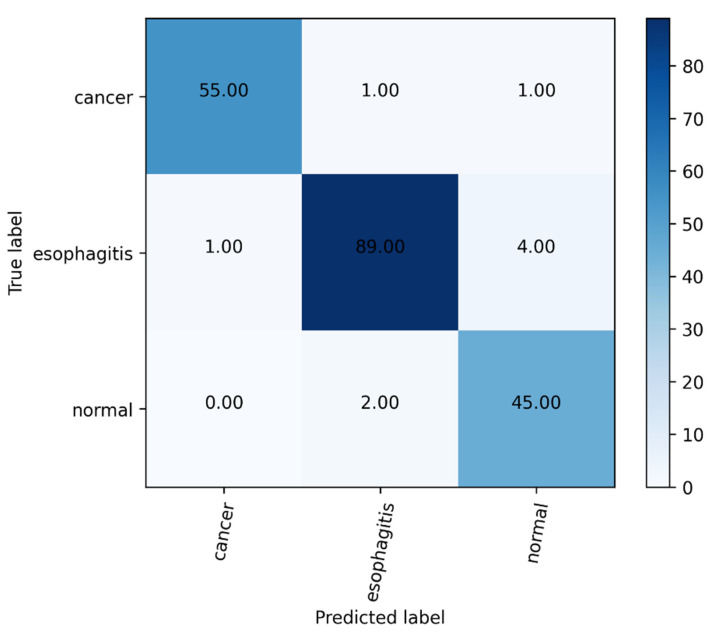
The confusion matrix of our model.

In this work, to solve the challenge of endoscopists in diagnosing esophageal lesions [55], we proposed a novel multi-task deep learning model to assist endoscopists in improving the diagnosis accuracy of esophageal lesions. The proposed model showed a favorable performance for diagnosing esophageal diseases, with an accuracy of 96.76%. We can also intuitively see from the confusion matrix in Figure 6 that most of the images in the test set can be predicted correctly. Furthermore, to further verify the clinical application value of the proposed multi-task deep learning model, endoscopists were asked to review every image of the validation dataset with and without using the proposed model. By using this model, the average diagnostic accuracy was increased from 83.84% to 90.57%. The improvements in diagnostic ability confirmed the feasibility of the proposed model for helping endoscopists discover lesions ignored previously.

On the other hand, the proposed model can mask the esophageal cancer region with a high Dice coefficient (71.27%) and IoU (82.47%). Although previous studies have applied deep learning to classify or segment esophageal diseases, deep learning models can seldom classify and segment esophageal lesions at the same time. To the best of our knowledge, this is the first such multi-task deep learning model developed in Macau.

The proposed multi-task deep learning model not only achieved high accuracy in esophageal lesion classification but also output the mask of esophageal cancer, thereby reminding the endoscopists to pay attention to the location of the suspicious lesion. We hope that the proposed model can be used in the following situations: during the examination, it finds and masks a suspicious area under WLI; this will prompt the endoscopist to use the NBI mode and perform a biopsy. We are currently developing the multi-task deep learning model based on WLI and NBI images to establish a more subjective method that combines the current white light algorithm with the NBI algorithm.

Our work has several limitations. First, since our datasets only come from Macau Kiang Wu Hospital, the sample size (including images in the training and validation datasets) was small. Therefore, we plan to collect more images of different esophageal types from different regions and invite more endoscopists to participate in our research. This will make up for the flaws of the imperfect data in this type of research. Second, our work only focused on cancer, esophagitis, and normal images and did not include other esophageal diseases such as esophageal polyps, esophageal leiomyoma, and ectopia of gastric mucosa. In the future, we will persistently collect these esophageal lesions and use them in the proposed model. Finally, we considered improving the robustness of the multi-task deep learning model for poor-quality images. The robustness of the multi-task deep learning model can be obtained by using poor-quality images during the training process. However, low-quality images will impair the convergence of the model and are not easily recognized by the model. Another feasible method is that we can use a model with a stronger learning ability to weaken the negative impact of low-quality images. We can also use a model with a stronger learning ability to weaken the negative impact of low-quality images. For example, we can consider the transformer [43] model that has recently shined in the field of deep learning or use NAS [56] to search for a specific model that deeply fits the endoscopic image of the esophagus. These methods will make the model more robust and able to cope with more complex situations.

## 6. Conclusions

In this paper, we constructed a multi-task deep learning model consisting of share layers and task-specific layers to achieve the classification and segmentation of esophageal lesions. The classification task determines the lesion type of the input image. Based on the classification task, image retrieval was used to provide more supporting information to endoscopists by finding a few samples that were the most similar in the input image. If the input image is cancer, the location of the cancer is further determined by cancerous area segmentation. To ensure the effectiveness and stability of the segmentation task, we developed an attention mechanism. The proposed model was evaluated on the testing set. The experimental results demonstrated that it was able to show a favorable diagnostic performance for classifying esophageal lesions with high accuracy and could achieve a high Dice coefficient and IoU for esophageal cancer segmentation. Furthermore, we invited endoscopists to compete with our model. The results showed our model achieved a classification accuracy of 96.76%. The accuracy of the endoscopists was 83.84%. Based on these promising results, the proposed multi-task deep learning model could become a potential assistant to help endoscopists in judging esophageal lesions.

## Figures and Tables

**Table 1:** Comparison of the methods for esophageal lesion classification.

Authors	Methods	Performance
Münzenmayer et al. [19]	content-based image retrieval	0.71 kappa
Riaz et al. [20]	autocorrelation Gabor features	82.39% accuracy
Yeh et al. [21]	color coherence vector	92.86% accuracy
Liu et al. [22]	support vector machines	90.75% accuracy
Nakagawa et al. [12]	SSMD	91.00% accuracy
Kumagai et al. [23]	GoogLeNet	90.90% accuracy
Liu et al. [23]	VGGNets, etc.	89.00% accuracy
Du et al. [25]	ECA-DDCNN	90.63% accuracy
Igarashi et al. [26]	AlexNet	96.50% accuracy

**Table 2:** Comparison of the methods for esophageal lesion segmentation.

Authors	Methods	Performance
Sommen et al. [29]	local color and texture features	0.95 recall
Yang et al. [30]	online atlas selection	0.73 DSC
Mendel et al. [31]	transfer learning	0.94 sensitivity
Huang et al. [33]	channel-attention U-Net	0.725 DV
Tran et al. [34]	spatial attention network and STAPLE algorithm	0.869 Dice
Chen et al. [35]	U-Net Plus	0.79 DV
Diniz et al. [36]	Atlas-based Residual-U-Net	0.8215 Dice

**Table 3:** Comparison of the classification results of our model and other models on the testing set.

Models	Top-1 Accuracy ± std	F1 Score ± std
VGG-16 [45]	92.68% ± 0.26	88.12% ± 0.26
ResNet-18 [46]	93.18% ± 0.25	88.36% ± 0.27
ResNeXt-50 [47]	94.34% ± 0.38	90.76% ± 0.33
Efficientnet-B0 [48]	95.15% ± 0.40	92.42% ± 0.39
RegNetY-400MF [49]	94.64% ± 0.52	91.57% ± 0.59
Ours	96.76% ± 0.22	94.22% ± 0.23

**Table 4:** The diagnostic performance of the endoscopists without and with the proposed model.

Performance		Accuracy	Precision	Sensitivity	Specificity	NPV	F1-Score
Our model	cancer	98.48%	98.21%	96.49%	99.29%	99.59%	97.34%
	normal	96.46%	90.00%	95.74%	96.69%	98.65%	92.78%
	esophagitis	95.96%	96.74%	94.68%	97.12%	95.28%	95.70%
	all	96.96%	94.98%	95.64%	97.70%	97.84%	95.27%
Endoscopists only	cancer	91.41%	87.04%	82.46%	95.04%	93.06%	84.69%
	normal	83.84%	60.87%	89.36%	82.12%	96.12%	72.41%
	esophagitis	76.26%	81.33%	64.89%	86.54%	73.17%	72.19%
	all	83.84%	76.41%	78.90%	87.90%	87.45%	76.43%
Endoscopists (single classification)	cancer	93.43%	95.83%	78.90%	98.58%	92.67%	87.62%
	normal	87.04%	65.67%	93.62%	84.77%	97.71%	77.19%
	esophagitis	81.31%	84.34%	74.47%	87.5%	79.13%	79.10%
	all	87.26%	81.94%	82.33%	90.28%	89.84%	81.30%
Endoscopists (our model)	cancer	96.46%	93.10%	94.74%	97.16%	97.86%	93.91%
	normal	90.40%	73.33%	93.62%	89.4%	97.83%	82.24%
	esophagitis	89.9%	96.25	81.91%	97.12%	85.51%	88.50%
	all	92.25%	87.56%	90.09%	94.56%	97.73%	88.22%

**Table 5:** The diagnosis results of the endoscopists after referring to the results of the proposed model.

Counts		Endoscopists (Before)		Total
		Right	Wrong	
Endoscopists (after)	Right	152	**27**	175
	Wrong	8	11	23
Total		160	38	198

**Table 6:** Comparison of the segmentation results of our model and other models on the testing set.

Models	IoU	Dice
U-Net [32]	63.55%	75.12%
PSPNet [50]	62.28%	75.62%
FCN [51]	63.95%	76.72%
Deeplab V3+ [52]	66.24%	78.20%
CCNet [53]	62.52%	74.90%
OCRNet [54]	61.04%	73.63%
SegFormer [44]	67.25%	80.38%
Ours	71.27%	82.47%
